# Aberrant KAT2A accumulations render TRIM22-low melanoma sensitive to Notch1 inhibitors via epigenetic reprogramming

**DOI:** 10.1186/s12967-023-04305-1

**Published:** 2023-07-06

**Authors:** Xiaoli Gu, Wei Min, Yibin Zeng, Ni Fan, Qihong Qian

**Affiliations:** Department of Dermatology, The First Affiliated Hospital of Soochow University, Pinghai Road 899, Suzhou, 215006 China

**Keywords:** TRIM22, KAT2A, Ubiquitination, Nocth1 signaling, Melanoma

## Abstract

**Background:**

Aberrant ubiquitin-proteasome system (UPS) triggers various disorders of biological events and contributes to progression of tumorigenesis. The tripartite motif containing 22 (TRIM22) was demonstrated to participate in the progression of multiple malignancies. Nevertheless, the role of TRIM22 in melanoma is still indefinite. This project aims to investigate the biological function of TRIM22 in melanoma and provide novel therapeutical targets.

**Methods:**

Bioinformatic algorithms were used to investigate prognostic significance of TRIM22. The in vitro or in vivo assays were used to explore the functions of TRIM22 in melanoma. The Co-Immunoprecipitation (Co-IP) and in vivo ubiquitination assays were used to assess regulations of TRIM22 on lysine acetyltransferase 2 A (KAT2A). The Chromatin immunoprecipitation (ChIP) assays and luciferase reporter assay were utilized to explore epigenetic regulations of KAT2A on Notch1.

**Results:**

Here, we utilized the bioinformatic methods to confirm that TRIM22 is decreased in melanoma than normal tissues. Patients with low TRIM22 levels had shorter survival months than those with high TRIM22 levels. Targeting TRIM22 favors melanoma cell migration, proliferation, and tumor development in vitro and in vivo. Mechanistically, TRIM22 interacts with KAT2A and promotes its degradation in a ubiquitination-dependent manner. Melanoma cells with TRIM22 deficiency depended on KAT2A to enhance malignant progression, including proliferation, migration, and in vivo growth. KEGG analysis determined the positive correlation between KAT2A and Notch signaling. Chromatin Immunoprecipitation (ChIP) assays implicated that KAT2A directly binds to the promoter region of Notch1 and mediates the enrichment of H3K9ac modification. KAT2A activates Notch1 transcriptional levels and sustains the stemness feature of melanoma cells. Nocth1 inhibitor (IMR-1) effectively suppresses the growth of TRIM22^low^ melanoma in vitro and in vivo but fails to inhibit TRIM22^high^ melanoma.

**Conclusion:**

Together, our study illustrates the mechanism by which the TRIM22-KAT2A-Notch1 axis promotes melanoma progression, and demonstrates that KAT2A/Nocth1 confers an epigenetic vulnerability in TRIM22^low^ melanoma.

**Supplementary Information:**

The online version contains supplementary material available at 10.1186/s12967-023-04305-1.

## Introduction

Skin cancer is a life-threatening malignancy worldwide and the morbidity is annually increased. Melanoma is the most aggressive subtype of skin cancer [1, 2]. As indicated by the authoritative data, the estimated number of new cases is 99,780, leading to nearly 7650 death in the United States during 2022 [3]. As is well-known, the ultraviolet is the main cause of cutaneous melanoma [4, 5]. According to the histopathological feature, melanoma has four types and the main type is superficial spreading melanoma [6, 7]. Multiple genetic alterations or abnormal biological events have been proved to participate in melanoma progression, including mitogen-activated protein kinase (MAPK) signaling, PI3K-AKT crosstalk, cell cycle, or epigenetic remodeling [8, 9]. Therefore, explorations of underlying mechanisms of melanoma would facilitate us to discover novel potential therapeutical targets. First of all, immune checkpoint inhibitors (ICIs) have been demonstrated to prolong the overall prognosis of advanced melanoma patients [10]. Secondly, BRAF and MEK inhibitors are investigated and appropriate for advanced BRAF-V600-mutant melanoma patients [11]. However, some patients still suffer from drug resistance and there is still a lack of efficient targets for advanced cases.

As is well documented, ubiquitination belongs to a protein post-translational modification type, which is dynamically regulated by ubiquitination-associated enzymes [12]. Dysregulation of the ubiquitination process regulates a wide range of biological processes, including autophagy, cell death, DNA damage repair, and metabolic disorders [13]. Intensive studies have implicated the essential role of ubiquitination in regulating tumor progression and highlighted the great therapeutic potential of targeting ubiquitination in various cancers, including melanoma [14]. For instance, Nadia Habel et al. revealed that FBXO32, a component of the SCF E3 ligase complex, controls transcription through the regulation of chromatin remodeling complex activity [15]. Baoshan Cai et al. found that USP5 attenuates NLRP3 inflammasome activation by enhancing autophagic degradation of NLRP3 in melanoma [16]. The tripartite motif-containing (TRIM) protein family is reported as a subfamily of the RING-type E3 ubiquitin ligase family [17]. Apart from the RING finger domain, TRIM family proteins contain one or two zinc-binding motifs, named B-boxes, and a related coiled-coil region. TRIM family proteins have been demonstrated to be involved in several biological processes, like transcriptional regulation, cell proliferation, apoptosis, and tumorigenesis [18]. TRIM22 is a member of the TRIM family of proteins and could be transcriptionally regulated by TP53. In gastric cancer, TRIM22 directly binds to Smad2 and inhibits the proliferation of cancer cells [19]. TRIM22 could also destabilize NRF2 to suppress osteosarcoma progression via activating ROS/AMPK/mTOR/Autophagy axis [20]. Apart from functioning as a tumor suppressor, TRIM22 could also enhance malignant features in various tumors. TRIM22 activates NF-κB signaling in glioblastoma by accelerating the degradation of IκBα [21]. However, the role of TRIM22 in melanoma progression has never been uncovered.

In this study, we report the down-regulation of TRIM22 in melanoma samples, which is associated with the prognosis of patients. TRIM22 inhibition enhances melanoma proliferation and migration. The KAT2A is a bona fide target of TRIM22, and TRIM22 deficiency promoted the accumulation of KAT2A proteins in melanoma. Together, our study revealed the novel role of the TRIM22/KAT2A axis in modulating melanoma progression and provided essential targets for treatment.

## Methods and materials

### Cell lines and culture

Human melanoma cell lines (A2058, MV3/14, A375, and C32) and embryonic kidney 293 T cells were from ATCC. 501Mel cell line was provided by Fudan University. All cell lines were used less than ten passages after STR profiling. Cells were cultured in Dulbecco’s Modified Eagle’s medium (DMEM) supplemented with 7% fetal bovine serum (FBS) and 1% penicillin-streptomycin.

### CCK-8 and colony formation assay

For MTT assays, 1000 melanoma cells were seeded in 96-well plates, and MTT (5 µg/ml, Sigma, USA) was added and incubated for 2 h at the indicated time. Formazan complexes were dissolved using 200 µl of dimethyl sulfoxide (DMSO; Sigma, USA) and the absorbance was determined at 450 nm. For the colony formation assay, medium supplemented with 0.6% agarose (1.5 ml) was placed in the wells of a 6-well plate as the supporting bottom layer, and 1000 cells in 1.5 ml of medium supplemented with 0.3% agarose were plated on the solidified substratum. After culture for 2–3 weeks, the colonies in each well were photographed and then imaged with a digital camera after being stained with MTT at 37 °C for 30 min.

### Migration assay

For the migration assay, cell migration was carried out using a Boyden chamber assay with 8 μm pore filter inserts (BD Bioscience). Cells (1 × 10^5^) were seeded on the upper chamber of a trans-well and RPMI + 10% FBS was placed into the lower chamber. Sixteen hours later, adherent cells to the underside of the filters were fixed with 4% paraformaldehyde, stained with 0.4% crystal violet, and counted. Results represent the average of triplicate samples from three independent experiments.

### Tumor sphere formation assay

Single-cell suspensions were plated at a distinct density of feasible cells in 96-well plates of ultralow attachment (Costar, Manassas, VA, USA). Sphere-formation culture has been conducted with slight changes. Cells were cultivated in a serum-free mammary epithelial growth medium (MEGM), supplemented by 1:50 B27 (Invitrogen, Grand Island, NY, USA), 20 ng/ml epidermal growth factor (EGF), 20 ng/ml basic fibroblast growth factor (bFGF) (BD, Franklin Lakes, NJ, USA) and 10 µg/ml heparin (Sigma, St. Louis, MO, USA). Spheroid figures were counted after 7–10 days. Primary spheres were gathered for in-vitro propagation, dissociated into a single cell suspension, and plated 96-well sheets in ultralow connection. The secondary spheroid figures were counted after 14 days of plating.

### Western blot assay

Cells were lysed in RIPA (CWBio, Beijing, China) containing protease inhibitors, on ice. The lysates were mixed with loading buffer and denatured at 100 °C for 10 min. The products were then subjected to sodium dodecyl sulfate-polyacrylamide gel electrophoresis (SDS-PAGE) and transferred to polyvinylidene difluoride (PVDF) membranes (Millipore, Billerica, Massachusetts, USA). The membranes were incubated with primary antibodies [TRIM22 (Abcam, ab224059), KAT2A (CST, ab217876), FLAG (Abcam, ab205606), Myc (Abcam, ab32072), Ub (Abcam, ab140601) and GADPH (Affinity, 1:1000)] and then the secondary antibody, which was conjugated to horseradish peroxidase (HRP;anti-rabbit IgG/anti-mouse IgG, CST, 1:5000). Immunoreactive bands were visualized using Image Lab after the addition of luminol-based chemiluminescent substrate (ECL; Millipore). The immunoblotting results were analyzed using Image Lab software.

### Immunohistochemistry staining

Tumor specimens were collected from patients diagnosed with melanoma. Fixed tissues were sectioned at a size of 4 μm. Tissue pieces were stained according to the manufacturer’s protocol with Biotin-Streptavidin HRP Detection Systems. Gently, sections were deparaffinized, and the antigen was repaired with sodium citrate and then incubated at room temperature with 3% hydrogen peroxide for 10 min. Sections of the tissue were blocked with goat serum at 37 °C for 15 min and incubated at 4 °C overnight with the specified vaccine. Positive and negative staining regions were identified using Image-Pro Plus 5.1 software (Media Cybernetics, Silver Spring, MD, USA). All patients have signed the informed consent, and the project is approved by the Ethics Committee of the First Affiliated Hospital of Soochow University.

### In vivo ubiquitination assay

For the polyubiquitinated KAT2A assay, we transfected the 501Mel cells with Myc-TRIM22 and Flag-KAT2A plasmids. Then, whole cell lysates prepared with RIPA buffer containing a proteinase inhibitor were subjected to IP using anti-Flag antibody to enrich KAT2A proteins. The levels of KAT2A ubiquitination were detected by immunoblotting with an anti-Ub antibody (Abcam, ab140601).

### Chromatin immunoprecipitation (ChIP)

After cells were fixed in 1% formaldehyde in PBS for 10 min, they were lysed and sonicated to achieve chromatin fragmentation. Then, the lysates were immunoprecipitated with KAT2A antibodies or negative control IgG (Cell Signaling Technology). The enrichment of the KAT2A protein with specific DNA fragments of the Notch1 promoter was measured by PCR. Input chromatin collected without immunoprecipitation was used as the positive control.

### Animal studies

Stably transfected human melanoma cells (1 × 10^6^) were subcutaneously injected into the right dorsal side of five-week-old female nude mice (n = 6). Tumor volume was monitored at the indicated time points. Two weeks after injection, the mice were sacrificed, and the tumors were excised, weighed, photographed, and subjected to immunohistochemical staining. All experimental procedures were approved by the Ethics Committee of the First Affiliated Hospital of Soochow University.

### Statistical analysis

A two-tailed Student’s t-test was used to evaluate the significance of differences between the two experimental groups. All results are presented as the mean ± SD, and *p* < 0.05 was considered statistically significant. All studies were performed with three to five technical and biological replicates. Expression correlation analysis was evaluated using the Pearson correlation coefficient. Survival analysis was evaluated by the Kaplan-Meier method and the log-rank test. Two-tailed student’s t test was used to compare the differences between two paired or unpaired groups. The Cox proportional hazards model was utilized to find prognostic factors based on the *survival* R package. The Shapiro-Wilk test was used to check the normal distribution. The non-parametric tests were used for data that are not normally distributed. All graphs were generated using GraphPad Prism 7.0 software.

## Results

### TRIM22 expressed lowly in SKCM samples that links a poor prognosis

**Fig. 1:**
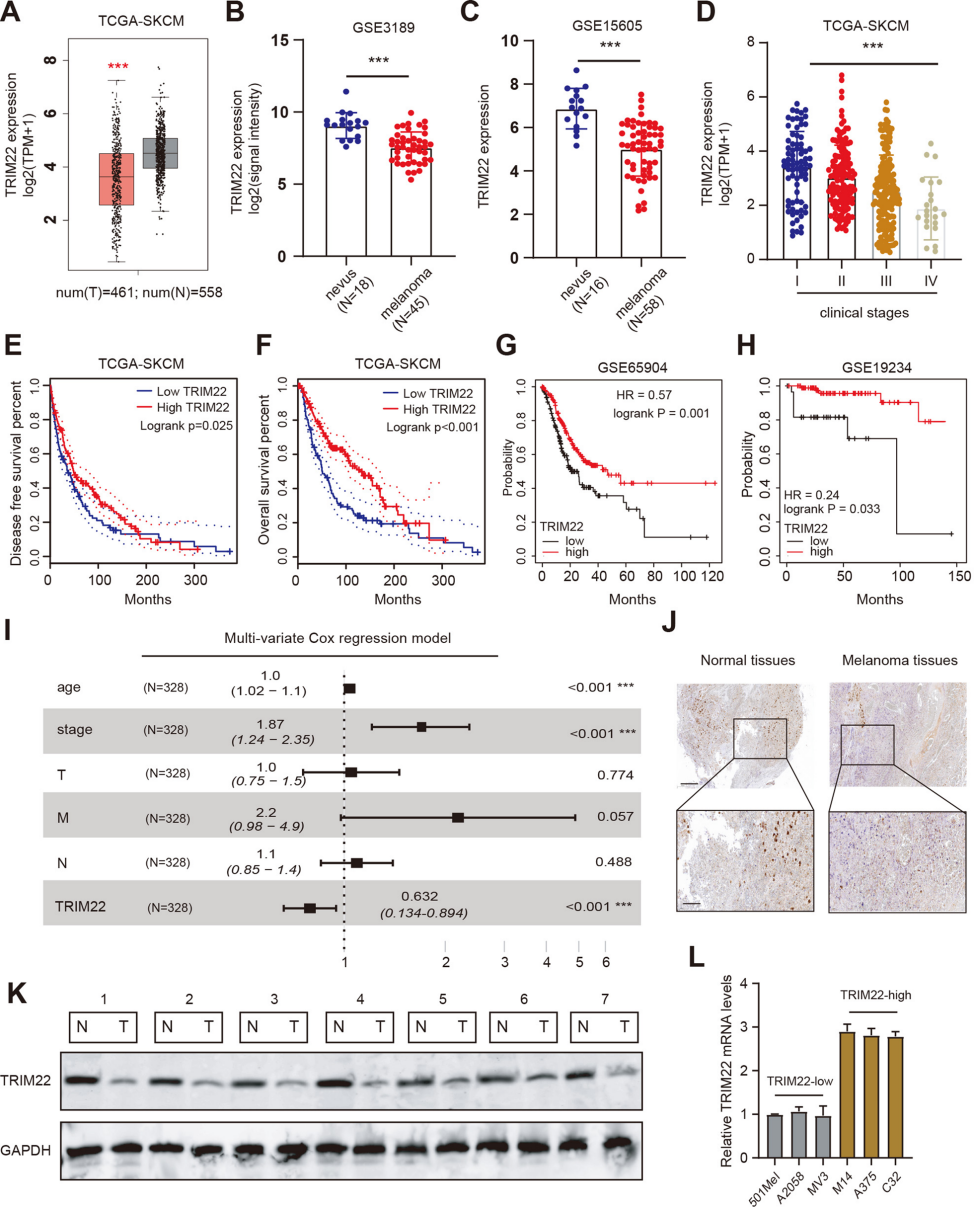
TRIM22 is down-regulated in melanoma samples. **A** The boxplot shows the differential expression levels of TRIM22 in melanoma and relative normal tissues. **B**, **C** Using other GEO datasets (GSE3189 and GSE15605), TRIM22 expression levels were found to be decreased in tumor samples relative to normal tissues. **D** Correlation analysis revealed the TRIM22 expression patterns in TCGA-SKCM patients with different clinical stages. **E–H** Kaplan-Meier analysis revealed that survival differences of TCGA-SKCM patients in low- or high-TRIM22 groups (E-F), GSE65904 (G), and GSE19234 (H). **I** Multi-variate Cox regression model was performed to confirm that low TRIM22 was an independent factor associated with the prognosis of melanoma patients. **J** Immunohistochemistry (IHC) was used to detect that TRIM22 expressed lowly in resected tumor samples relative to adjacent normal sections. **K** The 8 paired melanoma tissues and normal samples were used to compare the TRIM22 protein levels by western blotting assay. **L** The RT-qPCR assay was used to detect TRIM22 expression levels in a panel of SKCM cell lines and divided them into high and low groups. * *P* < 0.05, ** *P* < 0.01, *** *P* < 0.001. *T* tumor, *N* Normal

To assess the TRIM22 expressions in melanoma samples, we first downloaded the mRNA data of TRIM22 from the TCGA-SKCM cohort. Differential analysis suggested that TRIM22 was significantly down-regulated in tumors than that in normal samples (*P* < 0.0001, Fig. 1A, N = 558). In addition, we also downloaded the TRIM22 expression levels from the available microarray data (GSE3189 and GSE15605), and TRIM22 was decreased in tumor samples relative to that in normal samples with *P* < 0.0001 (Fig. 1B, C). We further collected the clinical characteristics of melanoma patients from TCGA-SKCM. The TRIM22 expression levels were found to be negatively associated with the clinical stages of patients (Fig. 1D). Furthermore, we categorized the melanoma patients into TRIM22-high and TRIM22-low groups according to the median data of TRIM22. Kaplan-Meier survival curve analysis with log-rank test indicated that patients with low TRIM22 levels had worse outcomes with shorter disease-free survival (DFS) months and overall (OS) survival months in the TCGA-SKCM cohort (Fig. 1E, F). We further demonstrated this prognostic finding in other validated datasets, including GSE65904 (HR = 0.57, log-rank test *P* = 0.0011) and GSE19234 (HR = 0.24, log-rank test *P* = 0.033) (Fig. 1G-H). Multi-variate Cox regression model analysis was performed in the TCGA-SKCM cohort by integrating age, clinical stage, TNM stages, and TRIM22 levels. The Forest plot revealed that the TRIM22 level was an independent prognostic factor for melanoma patients, as compared to TNM stages (Fig. 1I). We collected the melanoma patient samples and conducted the immunohistochemistry (IHC) assay. The results showed that melanoma tissues presented lower expression of TRIM22 while benign nevus showed higher expressions (Fig. 1J). Lastly, Western blotting assays were conducted to validate TRIM22 protein levels in 8 paired samples of tumor and para-cancerous tissues. Compared to adjacent normal tissues, TRIM22 protein levels were both down-regulated in melanoma sections (Fig. 1K). We further utilized the RT-qPCR assays to determine TRIM22^low^ (501Mel, A2058, MV3) and TRIM22^high^ (M14, A375, C32) SKCM cell lines (Fig. 1L). Lastly, these data implicated that TRIM22 was decreased in melanoma and low TRIM22 correlated with poor prognosis of patients.

### Down-regulated of TRIM22 promotes melanoma progression both in vitro and in vivo

**Fig. 2:**
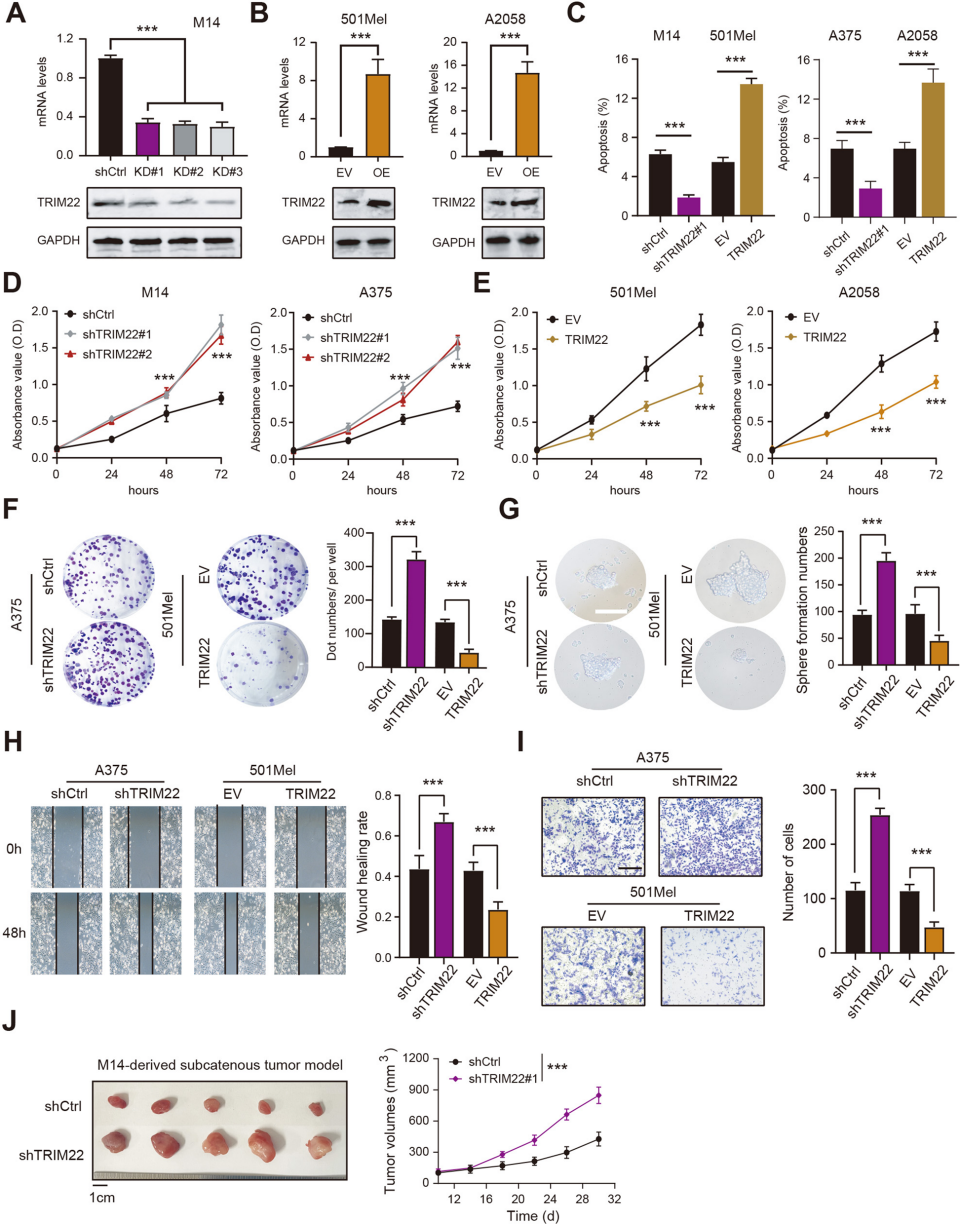
TRIM22 inhibition enhances melanoma progression in vitro and in vivo. **A** The RT-qPCR and western blot were used to detect the TRIM22 knockdown (KD) expression levels in M14 cells. **B** The western blot and RT-qPCR assays were used to confirm the efficiency of TRIM22 overexpression in 501Mel and A2058 cells. **C** The effect of TRIM22 inhibition or overexpression on melanoma cellular apoptosis was analyzed and quantified by flow cytometric assay. **D** Cell growth rates of M14 or A375 were enhanced by TRIM22 deficiency. **E** CCK-8 assays were used to confirm that cell growth rates of 501Mel and A2058 were suppressed in response to ectopic expression of TRIM22. **F** The colony formation assays were conducted in the indicated groups of A375 or 501Mel cells. **G** The sphere formation assays were performed in the indicated groups of A375 or 501Mel cells. The sphere numbers in each group were counted and compared. **H** The wound healing (WH) assays were performed in the indicated groups of A375 or 501Mel cells. **I** The invasion assays with quantified data were shown in the indicated groups of A375 or 501Mel cells. **J** The effects of TRIM22 knockdown on subcutaneous tumors were investigated in vivo assays. The quantified tumor volume curve was shown on the right. * *P* < 0.05, ** *P* < 0.01, *** *P* < 0.001

To further confirm the biological role of TRIM22 in melanoma, we transfected M14 cells with TRIM22-shRNA lentivirus to knock down TRIM22. The knockdown efficacy was confirmed by RT-qPCR and western blotting assays (Fig. 2A). Besides, we utilized the lentivirus technology to overexpress TRIM22 in 501Mel and A2058 cells (Fig. 2B). Firstly, we found that TRIM22 knockdown could notably enhance the apoptosis resistance of M14 cells. Conversely, TRIM22 overexpression significantly inhibited apoptosis resistance in 501Mel cells (Fig. 2C). CCK-8 assays further revealed that cell viability was significantly increased in the TRIM22-KD group compared to the control group (Fig. 2D). In contrast, TRIM22 overexpression attenuated the cell proliferation efficiency as compared to control cells transfected with EV (Fig. 2E). Meanwhile, we observed that low levels of TRIM22 increased, and ectopic expressions of TRIM22 decreased the colony formation abilities of melanoma cells (Fig. 2F). The sphere formation ability of A375 cells was enhanced in response to TRIM22 knockdown, whereas TRIM22 overexpression suppressed this effect (Fig. 2G). Intriguingly, wound-healing assay further suggested that TRIM22 knockdown enhanced the migrated rates of A375 cells, and TRIM22 overexpression suppressed melanoma cell migration (Fig. 2H). The invasive ability of A375 cells was enhanced by TRIM22 knockdown, while TRIM22 overexpression suppressed this effect (Fig. 2I). Last, of all, we also explored the in vivo roles of TRIM22 in melanoma. The xenograft assays suggested that TRIM22 knockdown remarkably enhanced in vivo tumor growth rates as compared to tumors from the control group (Fig. 2J). Collectively, these results show that TRIM22 downregulation promotes melanoma progression in vitro and in vivo.

### TRIM22 mediates the ubiquitination and degradation of KAT2A

**Fig. 3:**
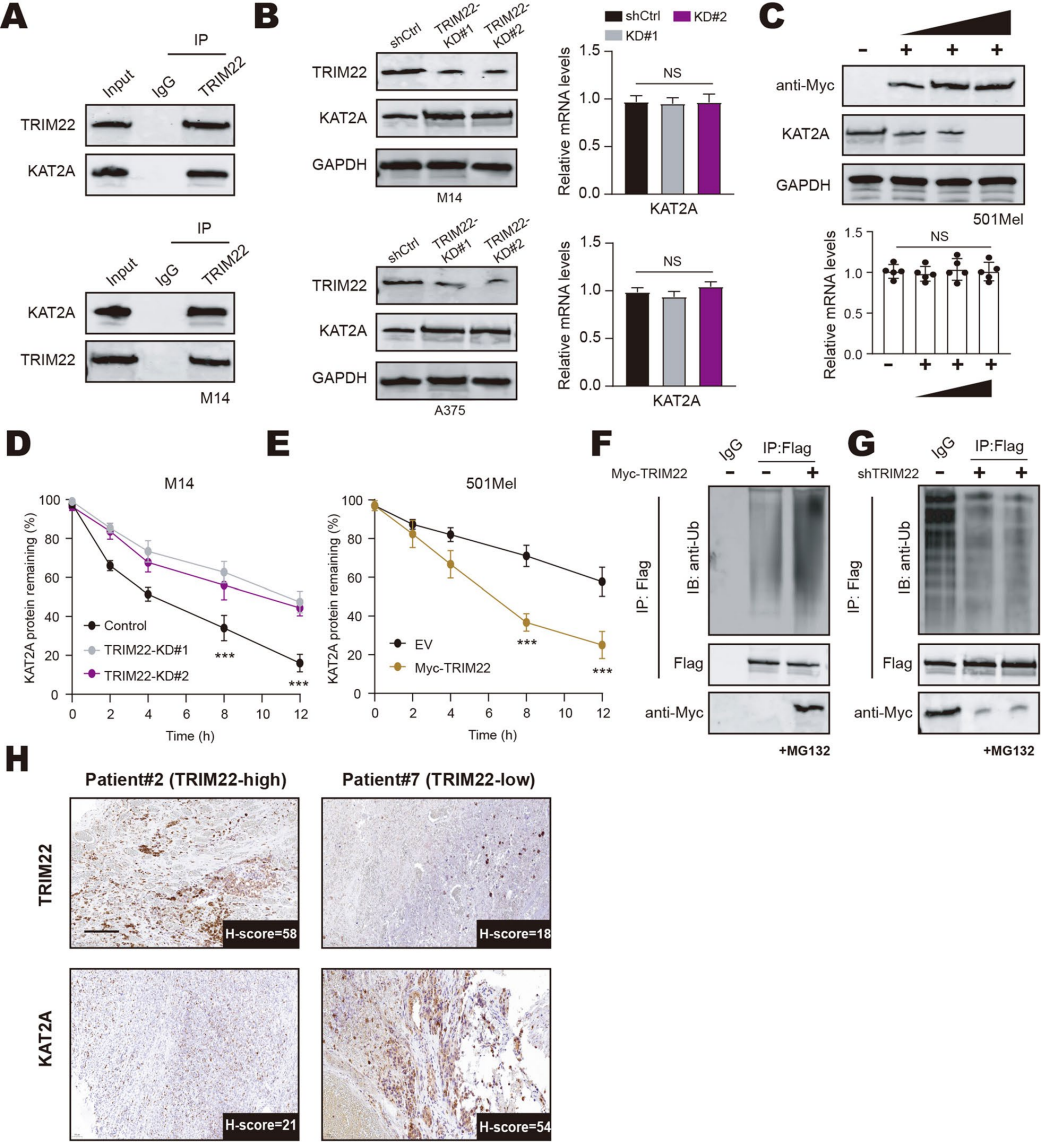
TRIM22 mediates the ubiquitination-dependent degradation of KAT2A proteins. **A** Co-Immunoprecipitation (Co-IP) assay was conducted to detect the endogenous interactions between TRIM22 and KAT2A from the cell extracts of M14 cells treated with 20 µM of MG132 for 8 h. **B** Western blot and RT-qPCR assays were used to detect the KAT2A expressions in A375 and M14 cells, individually. **C** Western blot and RT-qPCR assays were conducted to detect the mRNA and protein levels of KAT2A in 501Mel cells transfected with increased doses of Myc-TRIM22 plasmids. **D** The cycloheximide (CHX) assay was used to detect the half-life of KAT2A proteins in M14 cells. **E** The cycloheximide (CHX) assay was used to detect the half-life of KAT2A proteins in 501Mel cells transfected with Myc-TRIM22. **F** The in vivo ubiquitination assay was conducted in 501Mel cells transfected with the indicated plasmids and 20µM MG132 for 16 h. **G** The in vivo ubiquitination assay was conducted in TRIM22-KD M14 cells transfected with the indicated viruses and 20µM MG132 for 16 h. **H** The representative IHC graphs showed the converse relationships between TRIM22 and KAT2A proteins in collected melanoma tumors. * *P* < 0.05, ** *P* < 0.01, *** *P* < 0.001

To further investigate the underlying mechanisms by which TRIM22 exerts its function, preliminary exploratory results that TRIM22 could significantly immunoprecipitate KAT2A protein, one lysine acetyltransferase (Fig. 3A). Co-IP assays using anti-TRIM22 or anti-KAT2A antibodies confirmed the endogenous interactions between two proteins in M14 cells (Fig. 3A). In addition, we found that KAT2A protein levels were notably increased in TRIM22-KD M14 or A375 cells (Fig. 3B). However, KAT2A mRNA levels were not altered in response to TRIM22 depletion (Fig. 3B). Furthermore, we transfected 501Mel cells with stably increased doses of Myc-TRIM22 plasmids and observed the decreased, not mRNA, protein levels of KAT2A (Fig. 3C). Thus, TRIM22 could degrade KAT2A proteins in a dose-dependent manner. In line with the results, the cycloheximide (CHX) assays revealed that TRIM22 depletion led to a longer half-life of KAT2A proteins, as compared to those in control cells (Fig. 3D). In contrast, TRIM22 overexpression notably shortens the half-life of KAT2A proteins (Fig. 3E). As indicated in Fig. 3F, TRIM22 overexpression could promote the ubiquitination levels of KAT2A proteins. However, TRIM22 depletion could sufficiently suppress the ubiquitination levels of KAT2A proteins (Fig. 3G). Lastly, we also performed IHC analysis in the collected melanoma tumors and confirmed the negative relationships between the two proteins (Fig. 3H). Collectively, these data indicated that KAT2A is the bona fide substrate of TRIM22. TRIM22 mediates the ubiquitination and degradation of KAT2A proteins in melanoma.

### TRIM22 downregulation depends on KAT2A proteins to promote malignancy of SKCM

**Fig. 4:**
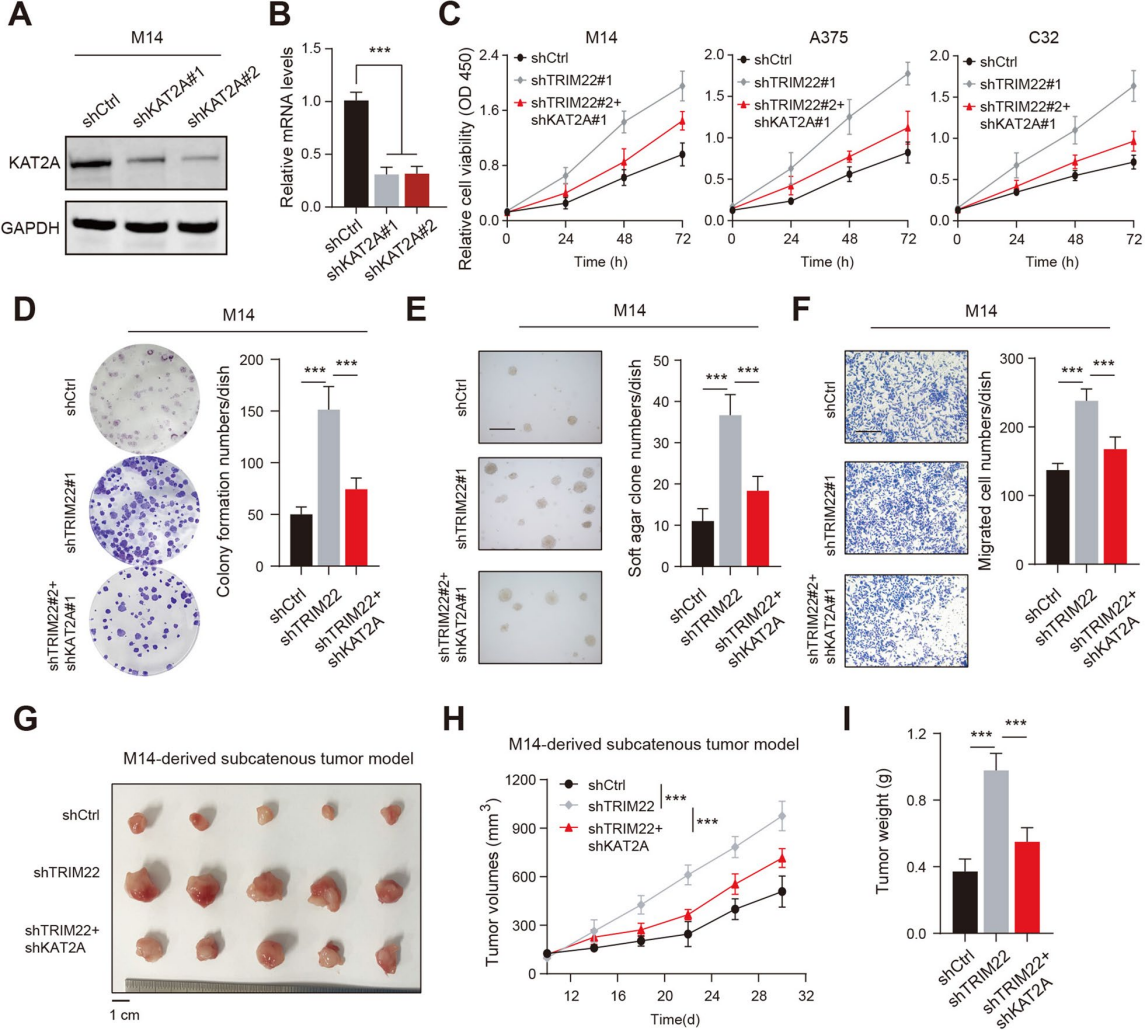
TRIM22 deficiency relies on KAT2A proteins to enhance the progression of SKCM. **A** The western blotting assay confirmed the KD efficiency of KAT2A in M14 cells. **B** The RT-qPCR assay detected the mRNA levels of KAT2A in control or KAT2A-deficient M14 cells. **C** The CCK-8 assays were used to detect cell growth rates of SKCM cells (M14, A375, and C32) from the indicated groups. **D** The colony formation assay was used to detect the colony formation ability of M14 cells derived from the indicated groups. **E** The soft-agar formation assay was used to detect the anchorage-independent growth in the soft agar of M14 cells. **F** The migration assay was used to confirm the migration properties of M14 cells from the indicated groups. **G** Knockdown of KAT2A suppressed TRIM22-KD-induced M14 cells subcutaneous tumor growth in nude mice. **H** The growth curve revealed the tumor volumes at the indicated time points from the three groups. **I** The tumor weight of tumors from indicated groups was quantified and compared. * *P* < 0.05, ** *P* < 0.01, *** *P* < 0.001

To further confirm the biological function of KAT2A in SKCM, we knocked down KAT2A in M14 cells using two different KAT2A short hairpin RNA (Fig. 4A, B). CCK-8 assays suggested that targeting TRIM22 enhanced SKCM cell growth (M14, A375, C32), which could be largely inhibited by KAT2A knockdown (Fig. 4C). In line with the findings, TRIM22 deficiency could significantly promote colony formation, soft-agar cell growth, and migration properties of SKCM cells, which could be largely abolished by KAT2A KD (Fig. 4D–F). Last, of all, the subcutaneous xenograft model confirmed that TRIM22 depletion led to enhanced in vivo tumor growth, which could be notably inhibited by KAT2A knockdown, as indicated by tumor volume curves and tumor weight (Fig. 4G–I). Taken together, our data implicated that TRIM22 deficiency depends on KAT2A to accelerate SKCM malignant features.

### KAT2A binds to the Notch1 promoter to activate the notch signaling

**Fig. 5:**
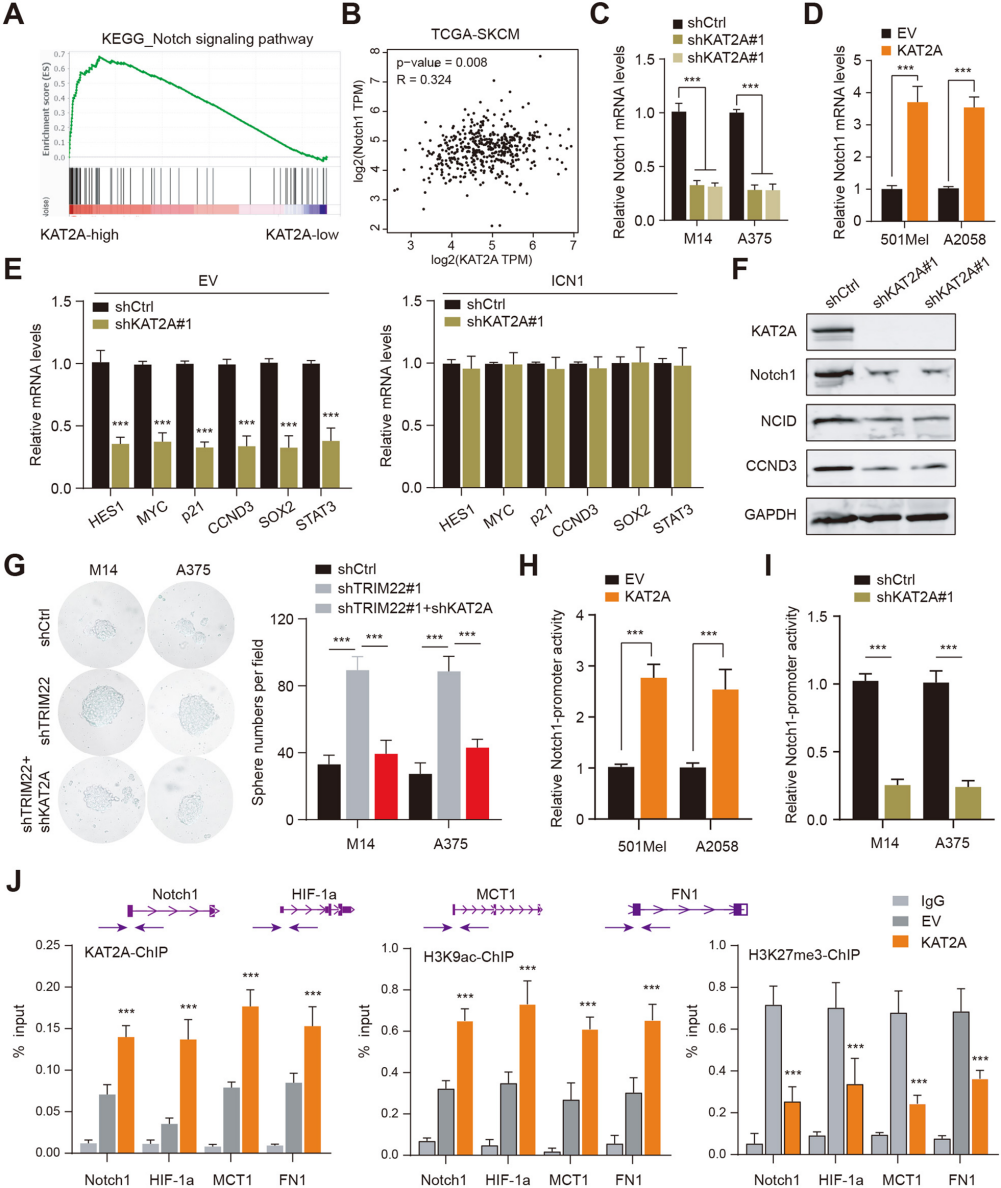
KAT2A binds to the Notch1 promoter to stimulate the Notch crosstalk. **A** GSEA revealed the tight associations between KAT2A and Notch1 crosstalk. **B** Correlation analysis was conducted between Notch1 and KAT2A mRNA levels in melanoma samples from the TCGA-SKCM cohort. **C** The RT-qPCR assay was used to detect Notch1 expression levels in M14 and A375 SKCM cells. **D** The RT-qPCR assay was used to detect Notch1 expression levels in 501Mel and A2058 cells. **E** The RT-qPCR assay was used to detect expression levels of Nocth1 downstream hits, and a rescue assay was conducted with ectopic expression of ICN1 in M14 cells. **F** The western blotting assay was used to detect expressions of Nocth1 downstream targets in KAT2A-KD cells. **G** Sphere formation assay and quantified data were shown in M14 or A375 cells from the indicated groups. **H** Luciferase reporter assay was used to detect the promoter activity by KAT2A overexpression on Notch1. **I** Luciferase reporter assay was used to detect the promoter activity by KAT2A KD on Notch1. **J** The ChIP-qPCR assays of KAT2A binding, H3K9ac, and H3K27me3 in genes, as indicated. Arrows indicate primer locations. * *P* < 0.05, ** *P* < 0.01, *** *P* < 0.001

KAT2A has been reported to promote transcriptional activity for certain histone or non-histone proteins through acetylation or post-translational modifications (Fig. 5A). We categorized the SKCM samples into TRIM22-high and TRIM22-low groups to perform bioinformatic analysis. Gene Set Enrichment Analysis (GSEA) revealed that Notch signaling was notably enriched in TRIM22-high samples relative to TRIM22-low samples (Fig. 5A). KAT2A and Notch1 mRNA expression levels were tightly correlated based on the TCGA-SKCM cohort (Fig. 5B). These data suggested that KAT2A was associated with Notch1 crosstalk. KAT2A knockdown significantly suppressed the Notch1 mRNA levels in M14 and A375 cells (Fig. 5C). Ectopic expression of KAT2A elevated Notch1 mRNA levels in 501Mel and A2058 (Fig. 5D). Then, we intended to ask whether the Notch pathway is an essential downstream pathway of KAT2A in SKCM. We performed the rescue assay by activating Notch signaling in KAT2A-KD M14 cells. Overexpression of the active form of Notch1 (Intracellular domain of NOTCH1 [ICN1]) could effectively promote the expression of NOTCH1 in KAT2A-KD M14 cells (Fig. 5E). Intriguingly, expressions of a series of Notch1 downstream hits were suppressed by KAT2A-KD, including HES1, MYC1, p21, CCND3, and SOX2. However, forced activation of the Notch1 by ICN1 could completely rescue the impaired levels of Notch1 downstream hit (Fig. 5E). In addition, we validated that KAT2A knockdown could suppress Notch signaling by western blotting assay (Fig. 5F). Given that Notch signaling is required for the maintenance of self-renewal capacities of cells, we thus demonstrated that TRIM22 deficiency depended on KAT2A to promote stemness features of SKCM (Fig. 5G). Mechanistically, luciferase reporter assay revealed that KAT2A could potentiate the transcriptional promoter activity of Nocth1 (Fig. 5H). However, KAT2A KD suppressed its promoter activity (Fig. 5I). The chip-qPCR assay revealed that KAT2A enhanced the H3K9ac enrichment on the promoter region of the Notch1 promoter and suppressed the binding of H3K27me3 (Fig. 5J). Taken together, these results implicated that KAT2A could enhance H3K9ac enrichment on the Notch1 promoter to elevate its expressions and activate Notch signaling in SKCM (Additional file 1: Fig. S1).

### TRIM22^low^ SKCM shows sensitivity to Notch1 inhibition (IMR-1)

**Fig. 6:**
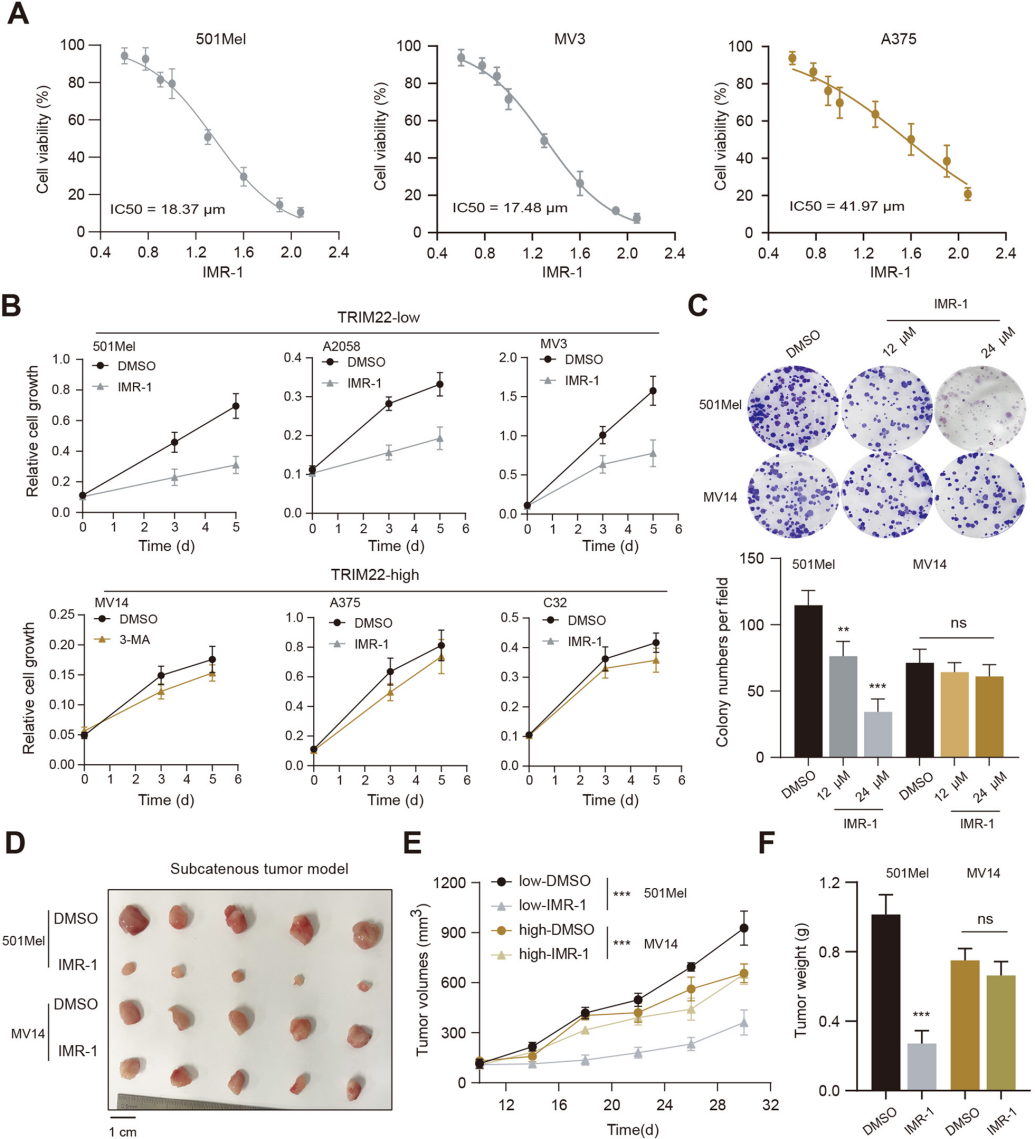
TRIM22^low^ SKCM is more sensitive to Notch1 inhibitor (IMR-1).**A** The IC50 data of TRIM22^high^ and TRM22^low^ cells were detected and the drug concentration curve was shown. **B** CCK-8 assays were conducted to compare the differences in cell growth with Notch1 KD between TRIM22^high^ and TRIM22^low^ cells. **C** The colony formation assay was conducted to compare the differences of IMR-1 in treating 501Mel (TRIM22^low^) and MV14 (TRIM22^high^) cells. **D** The representative subcutaneous tumor model was shown with tumors derived from TRIM22^low^ and TRIM22^high^ cells. **E** The tumor growth curve was quantified and compared with tumors in the indicated groups. **F** The tumor weight was quantified and compared with tumors from the indicated groups. * *P* < 0.05, ** *P* < 0.01, *** *P* < 0.001

**Fig. 7:**
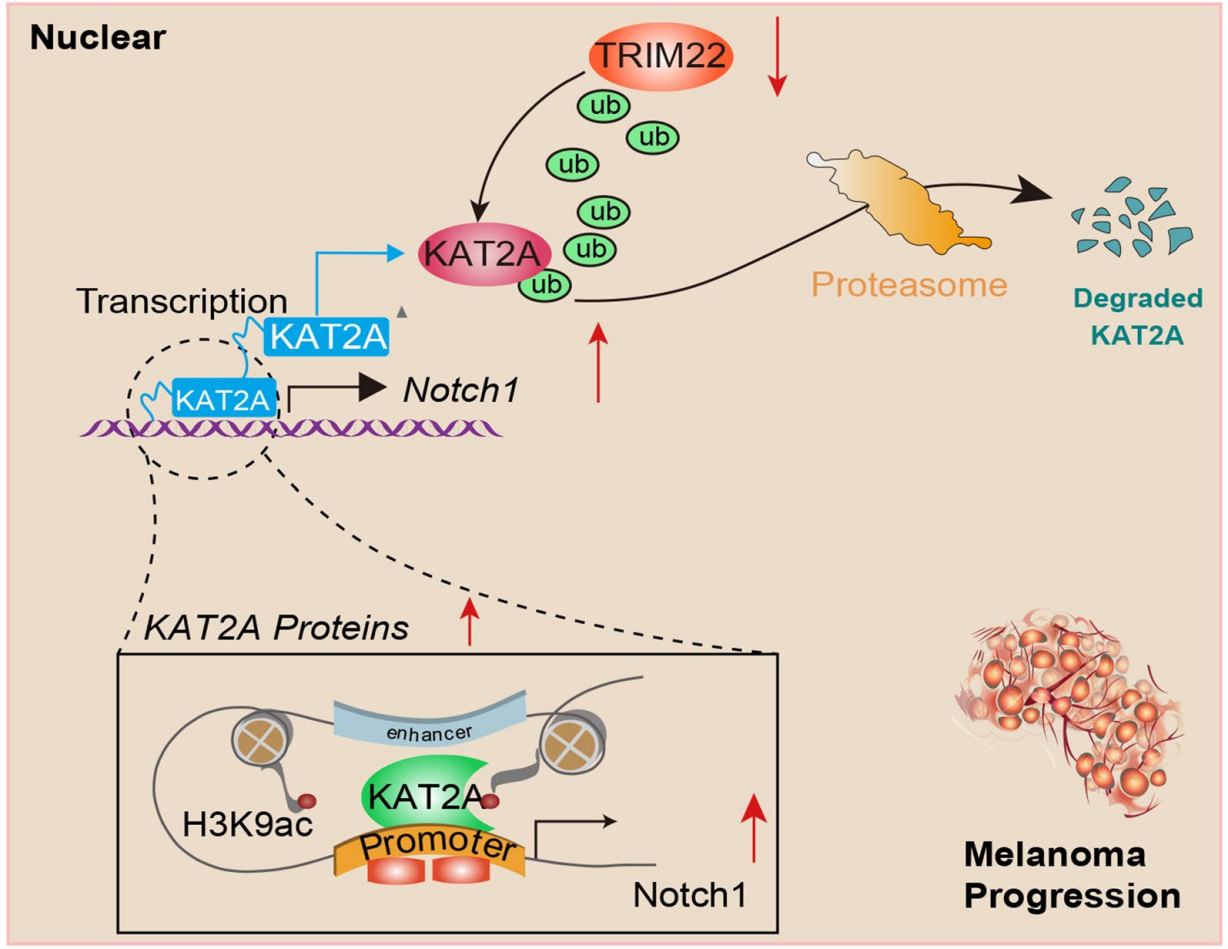
Graphical illustration showing the TRIM22-KAT2A-Notch1 axis in regulating SKCM stemness and progression

Given that tumor heterogeneity may influence drug responses of tumor cells, we thus considered whether TRIM22 expression levels could determine the sensitivity of SKCM cells to Notch1 inhibitors. We selected the TRIM22-high (M14, A375, C32) and TRIM22-low (501Mel, A2058, MV3) groups. In addition, we detected the half maximal inhibitory concentration (IC50) of IMR-1, one Notch1 inhibitor, in TRIM22-high and TRIM22-low cell lines (Fig. 6A). The IC50 of A375 (TRIM22-low cell) was 41.97 µM, while IC50 of 501Mel or MV3 was 18.37 µM and 17.48 µM. Thus, these data implicated that TRIM22-low SKCM cells were more sensitive to Notch1 inhibition. Then, CCK-8 assays revealed that IMR-1 could significantly inhibit the growth of TRIM22^low^ cells, while TRIM22^high^ cells exhibited mild differences in cell growth by IMR-1 (Fig. 6B). Likewise, addiction to Notch1 signaling of TRIM22^low^ SKCM cells was also demonstrated by colony formation assay, where IMR-1 could notably suppress cell growth potency of 501Mel cells in a dose-dependent manner, but not the MV14 cells (Fig. 6C). To further strengthen the in vitro findings, we also constructed the subcutaneous tumor model and tested the drug efficacy of IMR-1. Intriguingly, tumors derived from the 501Mel (TRIM22^low^) cells were more sensitive to Notch1 inhibition by IMR-1, but tumors derived from MV14 (TRIM22^high^) cells exhibited resistance to Notch1 inhibition, as evidenced by tumor volumes and tumor weight (Fig. 6D–F). Collectively, these findings suggested that IMR-1 is more appropriate for TRIM22^high^ cells, but not the TRIM22^low^ SKCM. Last of all, we illustrated the graphical mechanisms of the TRIM22/KAT2A/Nothch1 axis during the SKCM progression (Fig. 7).

## Discussion

Alterations in the DNA sequence have been found during melanoma progression, including chromosomal deletions or amplification, activating or inactivating mutations in genes [22]. Nevertheless, increasing attention has been attracted to epigenetic events in melanoma progression which could lead to stable inherited changes in gene expression without disturbing DNA sequences [23]. Containing histone modifications and DNA methylation, epigenetic events exert essential roles in determining cell identity, development, or differentiation [24]. Belonging to one type of post-translational modification, the E3 ubiquitin ligases are regarded to be the most essential and specific enzymes in the ubiquitin conjugation machinery [25]. In total, the E3 ligases are classified into four families according to the substrate binding domain: HECT-type, RING-finger-type, U-box-type, and PHD (plant homeodomain)-finger-type ligases [25]. Of note, TRIM protein has been explored to be part of cell proliferation and division, manipulate cell metabolism and apoptosis and be involved in tumor cell stemness and innate immunity [26, 27]. In the present study, we found that TRIM22 was down-regulated in melanoma samples relative to normal tissues, which was associated with poor prognosis of patients. Targeting TRIM22-induced melanoma progression, including cell colony formation, stemness, and migration capacities. Mechanistically, TRIM22 interacts with KAT2A proteins and mediates the ubiquitination of KAT2A. TRIM22 deficiency mediates the accumulations of KAT2A and depended on KAT2A to promote melanoma progression. KAT2A mediates the activation of Notch1 signaling to maintain the stemness features of melanoma. The ChIP-qPCR revealed that KAT2A promotes the H3K9ac enrichment on the promoter of Notch1 and decreased the H3K27me3 levels. Notch1 inhibitor (IMR-1) is effective to suppress TRIM22-low SKCM while exerting mild efficacy in TRIM22-high SKCM cells.

KAT2A belongs to a histone acetyltransferase that functions as a regulator of post-translational modification (PTM) process [28]. Besides, KAT2A acts as a succinyltransferase to succinylate H3K79 via binding to succinyl-coenzyme A (succinyl-CoA) [29, 30]. The succinyltransferase ability of KAT2A is indispensible for promoting tumor growth or migration [31]. In autoimmune disease, KAT2A could catalyze histone H3 lysine H9 acetylation to induce the IL2 expression and modulate T cell immunity [32]. Besides, KAT2A mediates the H3K79 succinylation to modulate β-catenin stability and aggravate the progression of pancreatic cancer [33]. In this study, we confirmed that KAT2A proteins are abnormally accumulated in TRIM22-low SKCM samples, not the mRNA levels. Targeting KAT2A could further suppress the SKCM progression. Bioinformatic analysis suggested that KAT2A could mainly activate Notch1 signaling, which has never been reported. We utilized the ChIP-qPCR to confirm the co-binding of KAT2A and H3K9ac on the promoter regions of Notch1. However, we are still uncertain about other essential candidates that could be manipulated by KAT2A. High-throughput sequencing technologies are warranted to confirm the genome-wide profile depicted by altered TRIM22 proteins.

As an evolutionarily conserved axis, Notch signaling mainly contains four receptors, like NOTCH1-4, and other various activating and suppressive ligands [34]. Notch signaling is reported to influence multiple biological events, like development, transdifferentiation, and cell fate [35]. Notch signaling is tightly associated with tumorigenesis or remodeling of tumor microenvironment [34, 36]. Yoshihiro Otani et al. revealed that NOTCH-induced immunosuppressive myeloid cell recruitment limited antitumor immunity in brain tumors [37]. Besides, NSD3-induced Methylation of H3K36 could also activate Notch signaling to promote breast cancer initiation and metastasis [38]. In melanoma, Zike Yang et al. implicated that Notch1 signaling in melanoma cells promoted tumor-induced immunosuppression via upregulation of TGF-β1 ^38^. In addition, Mitchell E Fane et al. suggested that reciprocal regulation of BRN2 and NOTCH1/2 signaling synergistically promotes melanoma cell migration and invasion [39]. However, the aberrant activation mechanisms of Notch signaling in melanoma are still uncertain. In this study, we report that KAT2A proteins could directly activate the Notch signaling via binding to the Notch1 promoter region. Notch1 inhibitor thus could be effective for TRIM22-low SKCM subgroups. However, the Notch1 inhibitor exerts limited efficacy for TRIM22-high SKCM due to suppressed KAT2A/Notch1 axis. As a result, the TRIM22/KAT2A axis might be predictive markers or indicators for the utilization of Notch1 inhibitors in SKCM.

However, this study still has some limitations that deserve to be further improved. First of all, we need more SKCM samples to look for the appropriate TRIM22/KAT2A cutoff to divide the patients. In addition, the other downstream candidates of TRIM22 contributing to SKCM progression are still unknown and deserved to be explored. Furthermore, aberrant activation of Nocth1 signaling in SKCM could lead to targeted drug resistance, which has not been well elucidated. Last of all, IMR-1 should be tested and validated in more pre-clinical SKCM models, including patient-derived xenografts (PDXs) or patient-derived organoids (PDOs).

## Conclusion

Taken together, our project suggested that down-regulated TRIM22 contributes to the malignant proliferation and metastasis of SKCM. TRIM22 mediates the ubiquitination-dependent degradation of KAT2A proteins. The altered TRIM22/KAT2A ubiquitination axis promotes SKCM stemness via activating Notch1 signaling. The Chromatin Immunoprecipitation (ChIP)-seq and Assay for Transposase-Accessible Chromatin with high-throughput Sequencing (ATAC-seq) analysis of KAT2A are warranted to be performed in the future to thoroughly determine the epigenetic roles of KAT2A in melanoma. Targeting Notch1 with IMR-1 could notably inhibit the growth of TRIM22^low^ SKCM, highlighting a therapeutical vulnerability for clinical translation. The SKCM Patients-Derived Organoids (PDOs) or Patient-Derived Xenografts (PDXs) models would be established in the future to determine the translational significance of our study.

## Supplementary Information

**Additional file 1.** The raw uncropped western-blotting graph used in the study.

## Data Availability

The data used to support the findings of this study are available from the corresponding author upon request. The TCGA-SKCM data was obtained from the TCGA platform via the GDC portal (https://portal.gdc.cancer.gov/).
