# Supplementary figures and images for: Aberrant KAT2A accumulations render TRIM22-low melanoma sensitive to Notch1 inhibitors via epigenetic reprogramming
Source: J Transl Med. 2023 Jul 6;21:443. doi: 10.1186/s12967-023-04305-1 (PMC10324160; doi:10.1186/s12967-023-04305-1)

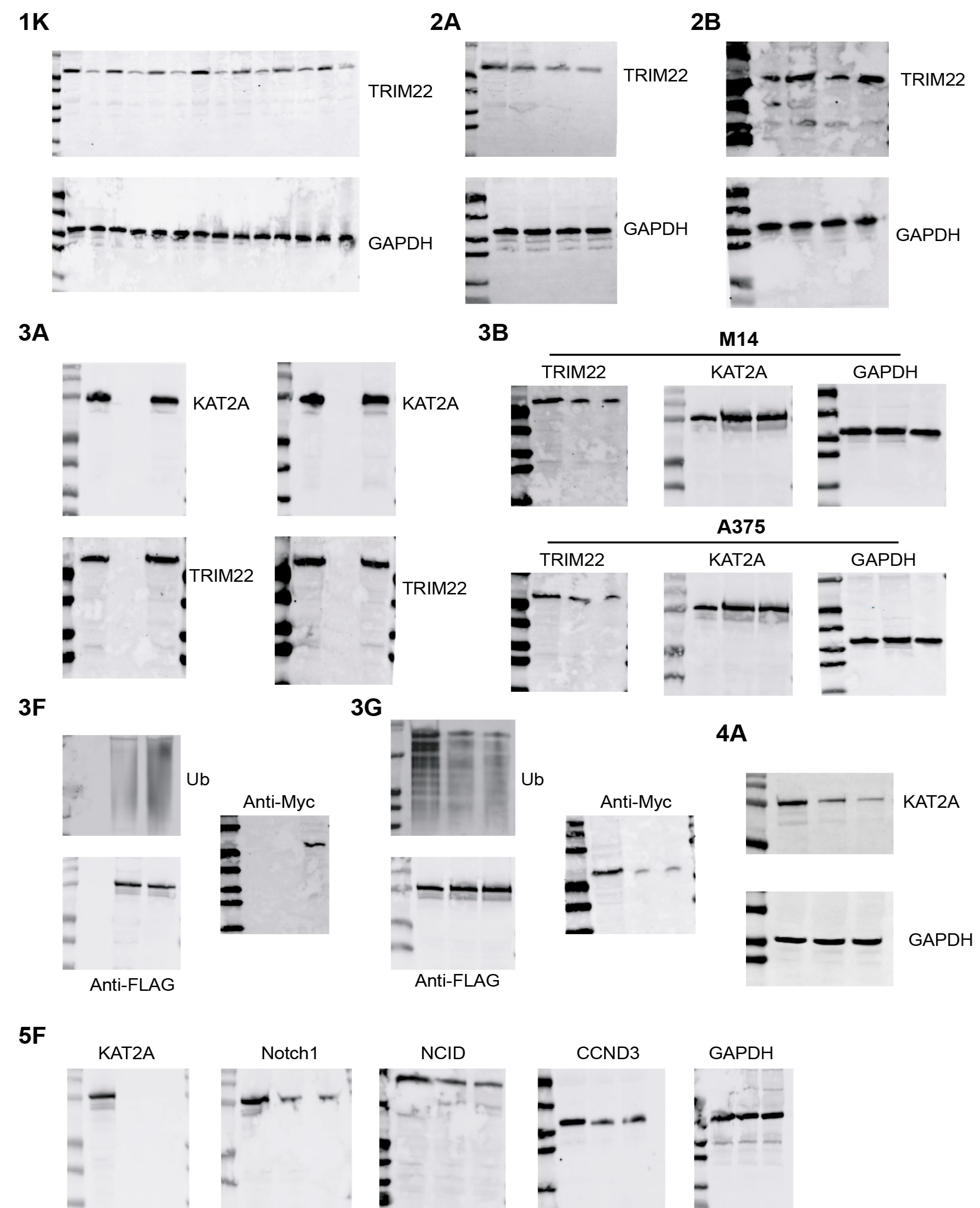

Supplement: Supplementary file 1 — Additional file 1. The raw uncropped western-blotting graph used in the study. [file 12967_2023_4305_MOESM1_ESM.jpg]
